# Effect of Despotic Leadership on Employee Turnover Intention: Mediating Toxic Workplace Environment and Cognitive Distraction in Academic Institutions

**DOI:** 10.3390/bs12050125

**Published:** 2022-04-27

**Authors:** Javed Iqbal, Ali Asghar, Muhammad Zaheer Asghar

**Affiliations:** 1 School of Education, Guangzhou University, Guangzhou 510006, China; javed@e.gzhu.edu.cn; 2 Dr Hasan Murad School of Management (HSM), University of Management & Technology, Lahore 54770, Pakistan; ali.asghar@umt.edu.pk; 3 Department of Education, University of Helsinki, 0014 Helsinki, Finland; 4 School of Doctorate, Education & ICT (e-Learning), Universitat Oberta de Catalunya, 08018 Barcelona, Spain; 5 Department of Education, University of Management and Technology, Lahore 54770, Pakistan

**Keywords:** despotic leadership, toxic workplace environment, cognitive distraction, employee turnover intention, structural equation modeling

## Abstract

Despotic leadership builds adverse emotions and turnover intentions in the employees of an educational organization. This study investigated the relationships among despotic leadership, toxic workplace environment, cognitive distraction, and employee turnover intention. This study is based on social exchange theory (SET), social psychology theories of behavioral intention formation (such as the theory of reasoned action and the theory of planned behavior), and of the despotic leadership style. A survey questionnaire containing 28 items was completed by 240 faculty members from four Chinese universities. The responses were documented on a seven-point Likert scale. We applied PLS–SEM (partial least squares structural equation modeling) to measure the effects. The outcomes showed that despotic leadership influenced employee turnover intention in academic institutions. Toxic workplace environment correlates with employee turnover intention. Cognitive distraction also correlates with employee turnover intention. Toxic workplace environment mediates the relationship between despotic leadership and employee turnover intention. Similarly, cognitive distraction mediates the relationship between despotic leadership and employee turnover intention. The study concluded that despotic leadership, toxic workplace environment, and cognitive distraction might increase employee turnover intention. This study adds to the literature in the field of despotic leadership, toxic workplace environment, cognitive distraction, and employee turnover intention in academic institutions. Furthermore, it offers valuable and practical implications along with recommendations for future research.

## 1. Introduction

Leadership is conceptualized as an ethical process that brings organizational justice, peace, and prosperity to all those involved [1,2]. Leadership develops and maintains a healthy and peaceful workplace environment where organizational members can learn, contribute, and remain while achieving common goals. The literature identifies various types of leadership behaviors and their different effects on organizational culture and employee turnover intention [3,4]. Despotic leadership builds adverse emotions and intentions. It negatively affects the organizational workplace environment and employee well-being and is positively associated with employee turnover intention [5].

Despotic leadership is characterized by undesirable characteristics such as arrogance, manipulation, and authoritarianism [6]. Despotic leaders concentrate on their own concerns and do not bother with their subordinates’ well-being in the organization [7]. Similarly, employees are endangered by despotic leadership as they are likely to show great respect to their superiors while having few precise mechanisms to accomplish their own tasks. Such conditions may raise their concerns regarding their capability of meeting their job objectives [6].

Employee turnover intention is among the critical concerns of educational institutions [7,8,9,10]. A high turnover rate disrupts institutional processes and functioning while also incurring costs in recruiting and developing new employees [7,8,9,10]. As per the relevant literature, the critical factors affecting employee turnover intention include salaries, psychological well-being, the mentoring system, an ethical climate, fair decision-making processes, job autonomy, and leadership behaviors. These factors significantly affect employee happiness and greatly impact their turnover intentions. The workplace environment is also an essential factor that affects organizational performance. A toxic workplace environment fosters unpleasant and painful incidents for individuals, eventually affecting their mental and physical well-being. Its effects are felt within the organization. However, owing to special motives, only a few employees actually register official complaints against such happenings. Such silence and avoidance behaviors create difficulties for researchers wishing to explore the topic [11]. It has been unanimously determined that the victims of workplace violence lack well-being. According to Maslow’s hierarchy of needs, security is a major concern for people in every situation, and uncertainty is extraneous to higher needs [12]. Thereby influencing their turnover intentions. 

Cognitive distraction is also a significant factor in employee turnover intention. However, studying cognitive distraction at the workplace has yielded vague results because distractions incur both costs and benefits [13]. Multi-tasking practices are being promoted at workplaces. Previous studies have determined how multi-tasking affects employee attention at the workplace. Interruptions and inconveniences among colleagues also cause cognitive distraction from ongoing tasks. A workplace analysis conducted by Czerwinski, Horvitz and Wilhite [13] indicated the means by which workers cope with the inconveniences raised by cognitive distraction in the sense of continual interruptions [14]. External interruptions for employees initiate a chain of cognitive distraction from the task at hand. This chain generally is comprised of four stages: diversion, realization, resumption, and retrieval [15]. Excessive cognitive distraction even after the resumption of an interrupted task, creates psychological stress among employees [16]. The reviewed literature offers clues concerning the impacts of despotic leadership on employee turnover intention. Such leadership stirs undesirable impressions and curbs employees’ social development. In this regard, an exploration of the effects of despotic leadership on employee turnover intention, especially regarding academic institutions, is much needed [5,7,17]. This study investigates the influence of despotic leadership on employees’ turnover This study investigates the influence of despotic leadership on employee turnover intention. In addition, the mediating effects of toxic workplace environment and cognitive distraction in the relationship between despotic leadership and employee turnover intention were also explored.

Most previous studies found the positive aspects of leadership style had an effect on employee job satisfaction [18]. According to studies conducted in industry and in different organizations, leadership is highly associated with employees’ psychological well-being, career growth, and performance [19]. Furthermore, various studies explored the positive influence of leadership styles such as servant leadership, authentic leadership, and transformational leadership [20]. That different leadership styles play an essential role in enhancing employee efficiency and job sustainability is a well-researched phenomenon [21]. It is, however, an understudied phenomenon in the traditional academic culture of eastern countries, where employees adopt silent and submissive behavior toward toxic leadership. Studies have also always reported on the good aspects of leadership while the dark side of negative leadership is still hidden. Therefore, how do despotic leadership and toxic workplace environment influence employee turnover intention in academia? There is still a lack of empirical studies on these topics [22]. This study adopted a relevant, worth-considering perspective offered by organizational leadership theory. The effect of despotic leadership on employee turnover intention has previously been studied through the organizational leadership [7,23,24]. However, the existing body of knowledge so far gives only a partial model of the relationships. Although it is empirically adequate and explains the role of organizational leadership, it does not comprehensively examine the phenomenon. This study attempts to bridge this gap through empirical investigation. Moreover, this perspective suggests that the adverse impacts of despotic leadership on employee turnover intention have not been thoroughly investigated.

Given these research gaps, this study’s primary goal is to investigate the impact of despotic leadership on employee turnover intention, which is explained in detail in the next section. This study also investigates the mediating roles of toxic workplace environment and cognitive distraction in the relationship. Various factors influencing the relationship between despotic leadership and employee turnover intention, including the factors of toxic workplace environment and cognitive distraction, as emphasized in the literature, were also considered in order to offer a wider perspective on this phenomenon. This study’s data was produced by a quantitative survey of 240 employees working at higher education institutions in China and used PLS–SEM to test the research hypotheses.

The present research paper offers numerous contributions. First, this study complements the current knowledge of the impact of despotic leadership on employee turnover intention and builds on the suggestions given by several studies in the recent literature. Second, this study investigates the effects of toxic workplace environment and cognitive distraction on employee turnover intention. Third, this study offers insights into the potential adverse effects of despotic leadership through the creation of toxic workplace environment and cognitive distraction in academic institutions on employee turnover intention. Fourth, this study suggests a synthesized research model that desegregates the corresponding viewpoints on despotic leadership’s direct and indirect impacts on employee turnover intention. Lastly, by conducting a comprehensive statistical analysis, it provides empirical evidence that reveals those effects and that aids in determining the concerns discovered so far in the literature. Overall, this research study offers practical understanding for academics, practitioners, and research societies.

**Figure 1:**
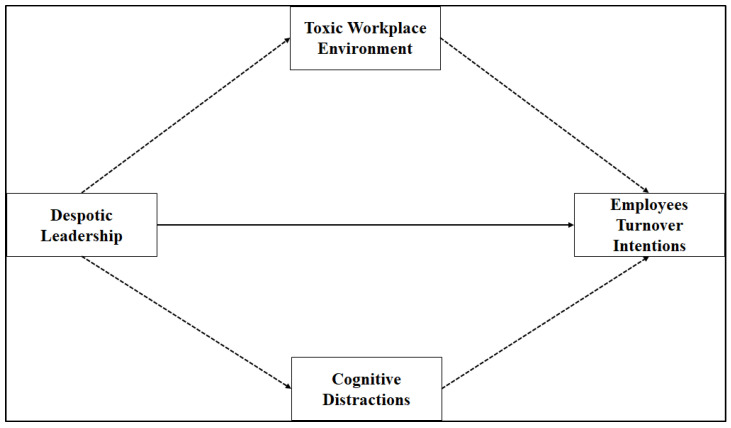
Research model.

According to the authors’ knowledge, this is the first study that explores the direct and indirect impacts of despotic leadership on employee turnover intention and examines toxic workplace environment and cognitive distraction as mediating variables. Built on these concepts, the research model examined in this study is presented in Figure 1. Thus, this study attempts to answer the subsequent three research questions (RQs):

RQ1: How does despotic leadership affect employee turnover intention in academia?
RQ2: How do toxic workplace environment and cognitive distraction affect employee turnover intention?
RQ3: How do toxic workplace environment and cognitive distraction mediate the relationship between despotic leadership and employee turnover intention?

The remaining parts of this study are designed as follows. Section 2 is comprised of a literature review and conceptual framework that focuses on the variables that explain despotic leadership, the toxic workplace environment, cognitive distraction, and employee turnover intention. Based on the literature review of organizational leadership theory, several hypotheses were formulated, as shown in the proposed research model (Figure 1). These hypotheses address the direct as well as indirect effects of despotic leadership, toxic workplace environment, and cognitive distraction on employee turnover intention. Section 4—research methodology—accounts for the explanation of the methodological processes as adopted by this study. Section 4 presents the results. Section 5 offers a discussion of the results and the conclusions drawn from them. It also includes implications, limitations, and suggestions for future research.

## 2. Literature Review

### 2.1. Despotic Leadership in China: A Background

Academic institutions in emerging nations are well aware of despotic leadership and its adverse effects. Several researchers discussed issues related to despotic leadership behavior in modern Chinese institutions. Liu, et al. [25] addressed the concept of despotic leadership conduct and employee turnover in a Chinese organization. As a topic, despotic leadership and its critical success factors are receiving much attention from scholars in China. The concept of despotic leadership emerged in the literature of organizational leadership in the 1970s in China through research conducted in Taiwanese organizations. Initially, it was considered to be one of the most essential elements of patriarchal leadership [26]. Despotic leaders exhibit an independent style and gain extensive consideration from administrators; therefore, their style has been explored in various contexts across the world [5,27]. The literature posits that the despotic leadership style is influenced by the Confucian value system, particularly in traditional Chinese families, where the father has ultimate control over family members, especially his children. In China, leaders in traditional organizations typically choose to behave as a father to impose a dominant central hierarchy. Despotic leadership behaviors and practices are common in Chinese institutions [28,29].

### 2.2. Theoretical Framework

We have based our research on three broader aspects of the theories; the first is the social exchange theory, the second is behavioral intention formation theories, and the third is leadership theory. 

Social exchange theory was used to explain the phenomenon of employee turnover intention. Behavioral formation theories (such as the theory of reasoned action and the theory of planned behavior) guided us in setting up hypothetical relationships between factors such as despotic leadership, toxic workplace environment, and cognitive distraction to explain employee turnover intention. Despotic leadership style theory highlighted the effect of the dark side of negative leadership and its impact on employees. 

#### 2.2.1. Social Exchange Theory (SET)

Social exchange theory (SET) initially explained the development and maintenance of interpersonal relationships. However, in the current literature, it has since been widely applied to explain the nature of the employee–employer/organization relationship [30]. Social exchange relationships develop when an organization or organization’s agent shows concern for its employees; this usually results in favorable consequences for the organization [31]. Social exchange theory (SET), widely used in human resource management and organizational behavior research, explains the employee–employer relationship regarding the reciprocity norm, which assumes mutual obligations between employees and employers [32]. For example, employees who receive favorable inducements from their organizations are more likely to have high organizational commitment [33], be loyal, and have fewer intentions to leave the organization [34]. Thus, practical employee attitudes and behaviors result from positive social exchange relationships. 

Social exchange theory suggests that an employee is more committed to his or her job and the organization if he or she perceives a just and balanced system of exchange [35]. In a perfect workplace scenario, workers perceive greater organizational support from colleagues and supervisors; in return, they offer the same support through positive behaviours and perceptions regarding the organization [36]. However, there may be some adverse effects on the progress of the mutual relationship between the employee and the employer if the former perceives that the organization gains from such a relationship but does not reciprocate within a period of time convenient to and expected by the employee [36]. Concerning experiencing fair reciprocity with the organization, employees would be more than willing to satisfy the employer’s expectations by exhibiting positive attitudes if they perceive that the organization trusts, recognizes, and values them [37]. Moreover, if employees observe that the organization provides more benefits than expected, then they perceive a positive balance in the employee–employer relationship, thereby encouraging them to carry out behaviors that are beneficial to the organization [38]. For example, an employer may provide recognition, compensation, career advancement opportunities, and job security in return for employee loyalty and efforts. Such dynamics portray the perception of fairness, which indeed exists if the interests of employees are protected and satisfied; as such, the perception of justice occurs as employees compare themselves with their co-workers and convey signals to their employers about the fair treatment that the organization provides [39].

#### 2.2.2. Behavioral Intention Prediction Theories

It is difficult to explain human behavior [40]. However, researchers [41,42] have tried to explain certain aspects of human behavior in terms of the satisfaction of various needs and desires. Intentions are defined as a person’s possible mindset in performing a certain behavior [41]. Human behavior is an outcome of an individual’s motives and intentions, which are influenced by social, personal, and situational factors [43]. This research focused on turnover intention rather than actual turnover because intentions are what lead a person to perform certain actions and behaviors. 

According to [44,45], personality traits, subjective norms, and attitudes, and situational factors and conditions play essential roles in determining the behavioral intentions of a person for a particular phenomenon. Still, it remains a question why a person behaves and acts in a specific way, and it is not an easy task to answer the posed question. Therefore, researchers have given different theories based on empirical evidence in order to understand intentions and human behavior. 

Ajzen and Fishbein [46] presented the theory of reasoned action (TRA) model. It is among the pioneering models from the field of social psychology for explaining intentions. This model presents a relationship between attitude, subjective norms, behavioral beliefs, intention, and personal behavior. TRA claims that all factors influencing human intentions are directed through attitude and subjective norms. Ajzen and Fishbein [46] described these variables as the exogenous factors of behavioral intention. These factors may be a political influence, the nature of a task, or an organizational structure [47]. TRA was an essential milestone in the development of another vital theory by Ajzen, the theory of planned behavior. 

Bandura [48] introduced the self-efficacy theory (SET). Ajzen refined the TPB to represent TRA. Ajzen proposed the addition of another determinant of behavioral intention, behavioral control, into the TRA model. TPB is considered a well-researched social and psychological theory for predicting behavioral intention in a situation where a person may lack control over behavior. 

By keeping in mind, the different intention formation theories mentioned above, we view turnover intention as a function of an external factor (i.e., despotic leadership), a sociological factor (i.e., toxic workplace environment), and a psychological factor (i.e., cognitive distraction).

The current study proposed a synthesized research model supposing that despotic leadership adversely influences employee turnover intention through toxic workplace environment and cognitive distraction in academic institutions. Despotic leadership, toxic workplace environment, and cognitive distraction have become serious concerns among scholars and academicians for the role of these factors in increasing employee turnover intention [5,14]. Despotic leadership works as a negative factor in organizational environments [49] and its effects on employee turnover intention were analyzed by researchers in the field of organizational leadership [7]. Organizational leadership theory contends that despotic leadership may significantly affect employee turnover intention. In academic environments, despotic leadership behavior affects organizational performance and creates circumstances that serve to increase employee turnover intention [25]. Therefore, the authors of this study assert that toxic workplace environment mediates the relationship between despotic leadership and employee turnover intention.

Furthermore, we seek to explore the mediated influence of cognitive distraction in the association between despotic leadership and employee turnover intention. This research analyzes these connections statistically and highlights despotic leadership’s influence on employee turnover intention through toxic workplace environment and cognitive distraction in an emerging nation. The present study also complements prior work in the field by illustrating the role of despotic leadership in creating the toxic workplace environment and cognitive distraction that leads to increased turnover intention.

It is well documented that academic institutions may exacerbate employee turnover intention by creating fears and toxicity in the workplace. Employee turnover intention has been defined as the plans workers have to leave an organization of their own volition [5]. However, employee turnover intention may also be assessed in the context of the organizational psychological environment. Despotic leadership in academia is very painful for subordinates and ultimately becomes the cause of their turnover intention [50]. This issue negatively impacts individual and institutional performance since universities must recruit, train, and retain new individuals in a constant cycle [25]. Employee turnover intention is a big challenge for organizational leadership, especially when employees work in a toxic environment that includes cognitive distraction. In such workplaces, employees may also want to leave. 

The existing literature explains that despotic leadership significantly influences employee turnover intention. Despotic leaders concentrate on their own interests at the cost of their subordinates’ well-being. Academic organizations are also in dire need of taking initiatives to maintain healthy and cognitive-distraction-free work environments in order to reduce employee turnover intention [14,23,51]. Researchers in the area of organizational leadership theory claim that employee performance and commitment to their respective organizations are based on the behaviors of their leaders [52]. Furthermore, employee productivity and creativity are damaged in a toxic workplace environment that features cognitive distraction, leading employees to want to leave their current jobs [50,53]. Based on this argument, the proposed research model of the current study is presented in Figure 1 and predicts the relationships in all hypotheses.

### 2.3. Despotic Leadership

Farh, Cheng and Chou [26] explain that despotic leadership has four common signs. First, despotic leaders rigorously control their subordinates as they desire their followers to obey their orders. Second, such leaders do not dare to accept ideas suggested by their subordinates. Furthermore, despotic leaders always claim recognition for successes and blame their subordinates for failures. Third, despotic leaders seemingly are always aware of whether others respect them enough. They control situations and benefit from others’ weaknesses. Fourth, such leaders are demanding, if not harsh, to their subordinates. They always remain dissatisfied with the work of their subordinates. They concentrate on assuming complete control over their subordinates in the great leadership style of the contemporary organizations in the era of socialism and thus increase productivity.

Furthermore, despotic leaders separate and absolutely dominate their subordinates and require them to obey orders categorically and completely [5,54]. The core of the dark side of leadership is despotic leadership This refers to the abuse of power and to self-interest Nauman, et al. [55]. Despotic leadership eventually negatively impacts organizational goals, morale, and employee satisfaction [56], as they are sacrificed for the leaders’ power, abuse, and self-interest [57]. 

### 2.4. Employee Turnover Intention

Employee turnover intention is referred to as the predisposition of employees to switch from their present places of employment and search for substitute jobs [8,9,10,50]. Employee turnover intention leads to actual turnover behavior [58,59], Turnover intention is influenced by external elements such as the accessibility of employment alternatives [60]. Employee turnover intention predications (and not just their behavior) allow institutions to predict how external and internal reasons may influence future turnover rates [9,61,62].

### 2.5. Toxic Workplace Environment

A toxic workplace environment features negative connections between employees and the workplace [63]. Workplace environment: the collaborative workplace environment and the toxic workplace environment. The collaborative workplace environment refers to a pleasant office environment featuring enjoyment, involvement, and citizenship behaviors. On the other hand, the toxic workplace environment features narcissistic conduct, aggressive and offensive leadership, antagonistic behavior from coworkers and bosses, and ostracism, harassment, and bullying [14]. Psychological and physical imbalance are observable features in a toxic workplace environment, where high levels of tension and exhaustion persist. Such environments become a source of employees’ mental and psychological health problems [14]. These factors stimulate counterproductive work attitudes in the workplace and ruin organizational productivity. Based on a thorough assessment of the literature and the philosophy of resource conservation, the current study focuses on some aspects of the toxic workplace environment, including ostracism, harassment, and bullying. Here, ostracism is described as the loneliness employees feel among their co-workers, family, bosses, and stakeholders [14,64]. Harassment refers to supervisors’, managers’, and peers’ inadequate handling and threats [65]. Bullying refers to the mistreatment of employees by individuals or a group in any circumstance [14]. The consequences of workplace ostracism, harassment, and bullying increase employee turnover intention and decrease feelings of job satisfaction [66]. The conservation of resources theory and an extensive review of related literature suggested these factors create toxic workplace environments and decrease employee performance and work engagement [63]. 

### 2.6. Cognitive Distraction

Cognitive distraction is anything that takes an operator’s mind away from some specific task [67]. When an operator is distracted, his or her attention floats from the task at hand. Distraction in the office falls into the behavioral and environmental dimensions integrated into the office environment [68]. Furthermore, employee productivity is based on a combination of individual and organizational characteristics [69]). In particular, the constant devotion of time to produce quality work is one of the main organizational elements that influences higher perceived productivity [69,70]. Researchers have also explored the physical aspects of a building: its lighting, temperature, and other facilities, all of which contribute to the overall office environment and affect productivity. On the other hand, less attention has been given to the study of cognitive distraction and employee productivity in organizational environments [70]. The literature suggests that institutions establish a wide range of workgroups to aid collaboration and that these might counter cognitive distraction. Workplace cognitive distraction can cost institutions millions of dollars in lost productivity. Creating a distraction-free work environment has major financial implications for employers, enhances employee well-being, and relieves frustration and stress [70]. 

## 3. Hypotheses Formulation

### 3.1. Despotic Leadership and Employee Turnover Intention

Employees of an organization, when demotivated or dissatisfied, intend to quit that organization [71]. Demotivation or dissatisfaction among employees lead to underperformance or employee turnover, which is undesirable for organizational leadership [49,72]. The literature highlights despotic leadership as a significant predictor of employee turnover intention [49,72]. One study examined the effects of despotic leadership on employee turnover intention in Pakistani organizations such as retail stores, factories, private banks, and two higher education institutions. The results revealed that despotic leadership behavior is a moderating construct, which, in a sense, is a predictor of employee turnover intention [7]. Based on the above literature, we developed the following hypothesis:

**Hypothesis** **1** **(H1).** *Despotic leadership positively influences employee turnover intention.*

### 3.2. Toxic Workplace Environment and Employee Turnover Intention

Several studies have investigated how a toxic workplace environment impacts worker psychology, resulting in poor behavioral outcomes [73]. Similarly, a toxic workplace environment decreases employee engagement and increases negative feelings among colleagues. Negative feelings include frustration and stress, which cause incivility. The literature shows that incivility, as an attribute of the toxic workplace environment, is among the predictors of employee turnover intention [7,14]. Another study explored workplace toxicity, job satisfaction, and employee turnover intention in organizations. The outcomes suggested that workplace incivility (toxicity) positively affects employee turnover intention and negatively affects job satisfaction [74]. This logic raises the following hypothesis:

**Hypothesis** **2** **(H2).** *A toxic workplace environment positively influences employee turnover intention.*

### 3.3. Cognitive Distraction and Employee Turnover Intention

Several studies have discussed the relationship between cognitive distraction and employee turnover [75,76]. Similarly, Gupta, et al. [77] posited that the connection between distractions or unnecessary interruption from supervisors provoked negative effects on task quality, ultimately affecting employee turnover intention. When it comes to job satisfaction, employee turnover intention was also examined in a separate study, one that also considered physiological aspects such as work–life conflict, regular working hours, and information technology [78,79]. Unfortunately, cognitive distraction in organizational settings has been underexplored by behavioral researchers, especially in higher education institutions. This research aims to address this gap in the existing body of knowledge by examining the role of cognitive distraction in developing employee turnover intention. The connection between cognitive distraction and employee turnover intention is assumed in the following hypothesis:

**Hypothesis** **3** **(H3).** *Cognitive distraction positively influences employee turnover intention.*

### 3.4. The Toxic Workplace Environment as a Mediator 

Several studies have investigated the connections between workplace environment, despotic leadership, and employee turnover intention in various contexts across the world [80]. The literature highlights the associations between despotic leadership and employee turnover intention [7,50]. Similarly, Ref. [23] explored employee turnover intention and employees’ senses of disengagement and disinterest, asking under what conditions despotic leadership could become less toxic for employees. Moreover, van Prooijen and de Vries [81] predicted the influence of despotic leadership on organizational conspiracy beliefs alongside its relations with conspiracy beliefs, which increased job insecurity and ultimately increased employee turnover intention. However, the role of a toxic workplace environment as a mediator seems to be less focused in the literature. This study intends to test this mediation and contribute to the body of knowledge. In this regard, the following hypothesis will be empirically tested:

**Hypothesis** **4** **(H4).** *A toxic workplace environment positively mediates the relationship between despotic leadership and employee turnover intention.*

### 3.5. Cognitive Distraction as a Mediator

One study explored the implications of employee turnover intention in the workplace and future research potential on the subject [82]. Similarly, Pereira, Müller and Elfering [75] examined workflow disruption and leadership-related social stressors to predict cognitive distraction or failure, factors that may contribute to the development of employee turnover intention. Another study looked at the relationship between despotic leadership and employee turnover. According to its findings, despotic leadership behavior is a mediating construct for good employee turnover intention [22]. Furthermore, the direct relationship between despotic leadership and employee turnover intention is a complex issue by definition [83]. This association could be mediated or moderated by several factors. This study aims to examine the effect of cognitive distraction in mediating the relationship between despotic leadership and employee turnover intention. The following hypothesis was prepared for empirical testing in this regard:

**Hypothesis** **5** **(H5).** *Cognitive distraction positively mediates the relationship between despotic leadership and turnover intention.*

### 3.6. Demographics and Turnover Intention

Previous studies [84] narrate that different physical and psychological traumas do not affect gender moderation for employment turnover intention. However, demographic moderators such as age [84] and experience Staniland, et al. [85] influence employee turnover. Another study also found the effect of teacher experience on turnover due to psychological stress [86]. Therefore, this study has also included age and experience as moderators of turnover intention.

**Hypothesis** **6a** **(H6a).** *The demographic of age has a positive moderating effect on turnover intention.*

**Hypothesis** **6b** **(H6b).** *The demographic of experience has a positive moderating effect on turnover intention.*

## 4. Methodology

The present study was conducted within the Chinese higher education framework, where somewhat fewer studies have already been conducted. It was stimulated by the need to explore how despotic leadership affects employee turnover intention in China. It may also impact the relationship among despotic leadership, toxic workplace environment, cognitive distraction, and employee turnover intention in academic institutions.

### Questionnaire Design

**Figure 2:**
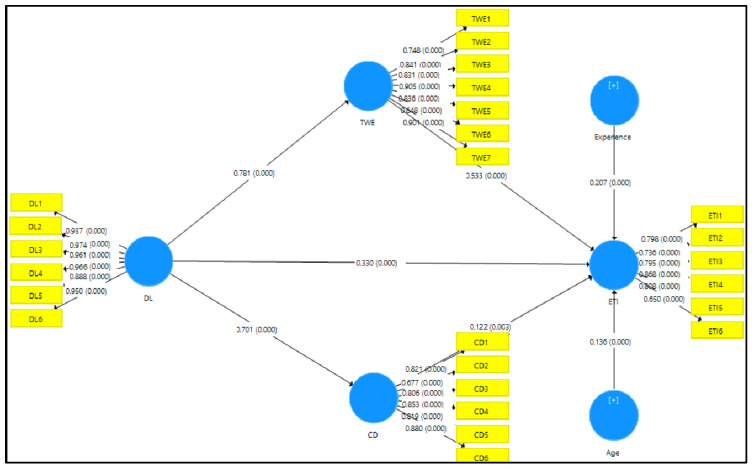
Research output model (structural relations among variables).

We collected the data using a survey questionnaire comprising 25 statements on a seven-point Likert scale (1–strongly disagree to 7–strongly agree). The statements were adapted from the literature related to despotic leadership, toxic workplace environment, cognitive distraction, and turnover intention. Despotic leadership was measured by responses to six statements. Toxic workplace environment was covered by seven statements. Cognitive distraction was comprised of six statements, and employee turnover intention consisted of six statements. The researchers conducted a pilot study with 25 participants having similar characteristics to the main sample to ensure the validity [87] and reliability of the instrument. Moreover, based on feedback, we revised a few of the statements to confirm that they were understandable to all participants. The details related to the questionnaire’s reliability and validity are presented in Tables 2 and 3 and Figure 2.

## 5. Measures

### 5.1. Despotic Leadership 

The seven items related to despotic leadership were adapted from Wang, Rasool, Zhao, Samma and Iqbal [29]. Examples of these items included: “The leadership in my organization is punitive and has no pity or compassion” and “The leadership in my organization expects unquestioning obedience of those who report to him/her.” Cronbach’s alpha value for despotic leadership was 0.916 (Table 2).

### 5.2. Toxic Workplace Environment

The eight items related to a toxic workplace environment were adapted from Rasool, Wang, Tang, Saeed and Iqbal [14]. Examples of these items included: “My supervisor/co-worker/subordinate never appreciates my physical appearance” and “My supervisor/co-worker/subordinate does not answer my greeting.” Cronbach’s alpha value for a toxic workplace environment was 0.917 (Table 2).

### 5.3. Cognitive Distraction

The six items related to cognitive distraction were adapted from Elsmore, et al. [88]. Examples of these items included: “I catch myself losing attention to the activity I am performing” and “I have to go back and check whether I did a task correctly.” The Cronbach’s alpha value for cognitive distraction was 0. 896 (Table 2).

### 5.4. Employees Turnover Intention

The eight items related to the employee turnover intention were adapted from Hanisch and Hulin [89]. Examples of these items included: “In the past year, I seldom completed my work assignment late” and “In the past year, it was very likely that I would have left my job if I received a suitable offer in another organization.” Cronbach’s alpha value for employee turnover intention was 0. 868 (Table 2).

## 6. Data Collection

Participants were chosen by using a random sampling technique, ensuring that all participants had an equal chance of being selected. We selected teachers from the faculties of higher education institutions from four universities in China. Pseudonyms were used for the selected universities. The formal survey stage began with randomly selected participants working in higher education institutions in China. This process was continued for almost 6 months. The questionnaires were distributed twice to test the reliability and integrity of the data. The first questionnaire comprised items related to despotic leadership, toxic workplace environment, and cognitive distraction in academic institutions. After 3 months, the participants were asked to fill out the questionnaires related to employee turnover intention from their current jobs, along with those related to the control variables that were measured. We used three channels of distribution for the questionnaires to make this process more specific. First, the authors distributed the questionnaires personally among the employees of the academic institutions. By doing this, we gave complete concept orientation to despotic leadership behaviors, toxic workplace environment, cognitive distraction, and employee turnover intention. Second, we received help from the deans and administrative staff in filling out questionnaires from the higher education institutions’ faculty members through the online questionnaire system. Third, we approached the faculty members with the help of our colleagues working as faculty members in selected organizations through the online questionnaire system. Participation in this study was entirely voluntary. Informed consent was received from all participants prior to final data collection, and detailed orientation was given to the participants regarding the research’s objectives and purposes. It clearly informed all participants that it would use the data only for academic research. In total, we circulated 400 questionnaires among the faculty members and received 240 usable questionnaires. The response rate was 67.5%. The total sample comprised 240 respondents (see Table 1).

### 6.1. Data Analysis

Both descriptive and inferential statistical analyses were applied in the present study. The researchers analyzed the data, applying measurement modeling and structural modeling through SmartPLS (version 3.3.3). First, we applied descriptive statistics to analyze the demographics of the participants included in the study. Second, we used the measurement modeling approach to gauge the reliability and validity of the instrument. In addition, descriptive analysis was performed for measuring the mean score and standard deviation of the constructs used in the structural equation modeling. Finally, structural equation modeling analysis was performed to test the relationships among the variables used in the research model. 

### 6.2. Demographics

The demographic distribution of the sample was as follows: as concerning gender, the respondents were 44.6% female and 55.4% male; in terms of age, those 30 years old and under comprised 30% of all participants, those 30–40 years old comprised 55%, those 41–50 years old comprised 10%, and those 51 years of age and above comprised 5%. This information is provided in Table 1.

#### Measurement Model

The measurement model was tested through CFA (confirmatory factor analysis) to measure the validity and reliability of the scales. The statistical software SmartPLS 3.3.3 was applied for measurement modeling and PLS–SEM. The SmartPLS is recommended for being least sensitive to sample size and more statistically efficient than other software packages that are used in covariance-based structural equation modeling approaches [90]. We applied structural equation modeling through SmartPLS to test the associations between the variables used in the research model. We measured the direct and indirect relationships among despotic leadership, toxic workplace environment, cognitive distraction, and employee turnover intention. Toxic workplace environment and cognitive distraction were used as mediating variables between despotic leadership and employee turnover intention. We ensured the validity and reliability of each construct before the start of the final data analysis. The reliability and validity of each construct were ensured through the measure modeling analysis approach before SEM analysis.

Factor loading, Cronbach’s alpha, rho_A, composite reliability, and AVE (average variance extracted) techniques were used. The threshold value for factor loading for each item was higher than 0.60. The threshold values for Cronbach’s alpha, rho_A, and composite reliability were higher than 0.70. Moreover, convergent validity was tested through AVE. The AVE value for all constructs should be above 0.5 [90,91]. Table 2 shows that the factor loading figures were above the threshold value of 0.6. The Cronbach’s alpha, rho_A, and composite reliability values were above 0.70. The AVE value was above 0.5, so it is concluded that the scale used in this study was reliable and valid (see Table 2).

Discriminant validity can be measured through two main approaches; Fornell and Larcker [92] and HTMT approaches in SmartPLS3.3.3 [90]. The researchers used the HTMT approach for measuring the discriminant validity of the reflective scales used in the research model. The HTMT approach is referred to as “a mean score value of the item correlations among the constructs related to the geometric mean of the average relationships among items evaluating the same construct” Henseler, et al. [93]. This approach is more valid and reliable in SEM analysis as compared with others, such as the Fornell and Larcker [92] approach, which was criticized by Henseler, Dijkstra, Sarstedt, Ringle, Diamantopoulos, Straub, Ketchen Jr, Hair, Hult and Calantone [90]. Fornell and Larcker’s approach is not strong enough to produce efficient results as compared with HTMT. Henseler, Dijkstra, Sarstedt, Ringle, Diamantopoulos, Straub, Ketchen Jr, Hair, Hult and Calantone [90] suggested a threshold value less than 0.90 for HTMT. A value above 0.90 for HTMT suggests the presence of discriminant validity issues. The results in Table 3 reveal that the HTMT value for each construct was less than 0.90. Thus, the scale fulfilled the requirement of discriminant validity. 

The structural equation modeling technique generally assures the elimination of the problem of collinearity among dimensions through the variance inflation factor (VIF). The threshold value for VIF is less than 5. A VIF value above 5 indicates that there is a collinearity problem present between dimensions [91]. In this study, the value of VIF was less than 5, ranging between 1.000 and 3.567 and indicates that no collinearity problem exists among the dimensions. 

Harman’s one-factor analysis helps in finding out the common method bias [94]. We conducted a Harman one-factor test to address the CMB issue. We applied factor analysis to all constructs as one principal component factor. Researchers Podsakoff, et al. [95] recommend that the result of un-rotated factor analysis must be less than 50%. Results of Harman’s one-factor analysis showed 36.24%. Therefore, the factors of our study showed no issues with common method bias. 

Model fit is measured based on the three most commonly used indicators: SRMR, NFI, and RMS_theta in SmartPLS. The threshold value for SRMR is from 0 to 1, and less than 0.80 is considered ideal for a good model fit [96]. The NFI range is between 0 and 1, and a figure greater than 0.90 is deemed suitable for the appropriateness of the overall model [96,97]. The RMS_theta is the most suitable indicator for assessing reflective measurement models, and the threshold value for a good model fit is less than 0.12 [90]. In this study, the value of SRMR was 0.079, which is less than 0.80. The value of NFI was 0.87, and it is less than 0.9. It reveals little difference. The RMS_theta value was 0.121—also not much higher than the ideal value, and as such, it is also appropriate. Thus, it is concluded that the model of this study is reasonably well-fitted in general. The analysis of collinearity and model fit are presented in Table 4.

In SEM analysis, we assessed the explanatory power of the output model with a range of R^2^ between 0 and 1. A value close to 1 is appropriate for higher explanatory power. The threshold values of R^2^ reach 0.25, 0.50, and 0.750, which are respectively considered to indicate weak, moderate, and strong explanatory power [91]. Table 5 indicates the explanatory power of toxic workplace environment, cognitive distraction, and employee turnover intention as 0.609, 0.491, and 0.828. Both workplace environment and employee turnover intention dimensions have a strong degree of explanatory power. However, the cognitive distraction dimension has a moderate level of explanatory power. Therefore, the model in this study explains that the latent variables are appropriate in terms of explanatory power. 

### 6.3. Descriptive Analysis

Table 6 explains the descriptive statistics of the survey respondents. The 7-point Likert scale was applied to record the responses. A total 240 responses were usable. The range of mean values for all responses was from 4.273 to 5.472. The range of standard deviations fell between 1.263 and 1.336. Details concerning the descriptive statistics of the survey respondents are revealed in Table 6. 

### 6.4. Structural Equation Modeling

SEM analysis was applied through SmartPLS 3.3.3 with the bootstrapping technique (5000). This study used this technique to measure path estimates, *p*-values, t-values and confidence intervals [98]. Direct and indirect relationships were measured among the constructs used in the research model. The analysis results showed that despotic leadership had a significant association with employee turnover intention (β = 0.330, *p* < 0.05) and affirms hypothesis H1. Similarly, a toxic workplace environment significantly connected employee turnover intention (β = 0.533, *p* < 0.05), which affirms hypothesis H2. Furthermore, cognitive distraction also showed a significant connection with employee turnover intention (β = 0.122, *p* < 0.05), which affirms hypothesis H3. Furthermore, we also measured two control variables, namely age and experience, and both significantly influenced employee turnover intention (β = 0.136, *p* < 0.05; β = 0.207, *p* < 0.05). The analysis results showed that age had a significant association with employee turnover intention (β = 0.136, *p* < 0.05) and affirms hypothesis H6a. The analysis results showed that experience had a significant association with employee turnover intention (β = 0.207, *p* < 0.05) and affirms hypothesis H6b. Table 7 and Figure 2 present more details.

In this study, we measured the indirect relationship of despotic leadership with employee turnover intention, while a toxic workplace environment was used as a mediating variable. Table 8 presents the results. Toxic workplace environment mediated the connection between despotic leadership and employee turnover intention (β = 0.416, *p* < 0.05); therefore, hypothesis H4 was accepted. Similarly, cognitive distraction mediated the association between despotic leadership and employee turnover intention (β = 0.086, *p* < 0.05). Thus, hypothesis H5 was affirmed. The results indicate that the hypotheses listed in Table 8 are affirmed. Figure 2 also shows the path coefficients of the model.

## 7. Discussion

We investigated the impact of despotic leadership on employee turnover intention in Chinese academic institutions with the mediating effects of toxic workplace environment and cognitive distraction. As a result of this investigation, a synthesized research model for the present study was implemented and revised. Similar prior studies have been carried out in developed nations but very few have examined such issues in emerging nations [5,73]. There is an urgent demand for such research in the field of higher education as it is essential for reducing employee turnover intention owing to despotic leadership behavior in academic institutions in China. As per the authors’ knowledge, the current research was among the first to measure the impact of despotic leadership on employee turnover intention in Chinese academic institutions, mainly using toxic workplace environment and cognitive distraction as mediators. We discuss the study’s findings in Section 7.1 and Section 7.2.

### 7.1. Theoretical Addition

This study explored the direct connection between despotic leadership and employee turnover intention and established that despotic leadership significantly affected the latter, affirming our intuition in hypothesis H1. The study results were consistent with previous studies’ findings that showed that despotic leadership impacted employee turnover intention. Similarly, Albashiti, Hamid and Aboramadan [80] conducted a study to explore the connections between despotic leadership and employee turnover intention. Those results revealed that despotic leadership had adverse effects on employee turnover intention in the hospital industry. Thus, the present research validates the notion that despotic leadership and employee turnover intention persist in Chinese academic institutions. It shows that despotic leadership influences employees’ intentions to leave academic institutions. 

The present study’s analysis of the direct relationship between the toxic workplace environment and employee turnover intention demonstrated that the former significantly impacted the latter, supporting our intuitions in hypothesis H2. The present study results are consistent with previous assertions that a toxic workplace environment affects employee turnover intention among university students [99]. Accordingly, Anjum, et al. [100] posited that a toxic workplace environment could increase employee turnover intentions in an organization. This study confirms the findings of previous studies that show that toxic workplace environment has a direct relationship with employee turnover intention. Universities are sensitive places where intellectuals, philosophers, and researchers are busy in producing creativity, innovation, and research. A toxic work environment develops academicians’ intentions to leave the toxic place of their intellectual work. 

The present study explored the direct connection between cognitive distraction and employee turnover intention. Its results revealed that cognitive distraction has deep-rooted effects on employee turnover intention, which supports our intuitions in hypothesis H3. These results are supported by previous studies’ assertions that incivility and cognitive distraction stimulate employee turnover intention [101]. Correspondingly, Shaukat, et al. [102] recommended exploring the association of cognitive distraction with job burnout in future studies. Our study investigated this construct and found a positive relationship between cognitive distraction and employee turnover intention in Chinese academic institutions, affirming the prior study’s assumptions. Therefore, cognitive distraction directly influences the development of academicians’ intentions to leave their organizations. 

The current study also measured how toxic workplace environment mediated the connection between despotic leadership and employee turnover intention. The outcomes of the present study established that toxic workplace environment positively mediated that association, thus supporting H4. This result validated the research findings of Malik and Sattar [103] who found that despotic leadership, in tandem with a toxic workplace environment, increased employees’ turnover intentions. Similarly, Albashiti, Hamid and Aboramadan [80] investigated psychological distress, which is alternatively used to represent toxic workplace environment, as a mediator between despotic leadership and employee turnover intention. Therefore, the results of the present study indicate that an indirect positive relationship exists between despotic leadership and employee turnover intention, as mediated by toxic workplace environment in Chinese academic institutions. We may conclude that despotic leadership produces a toxic workplace environment which is not suitable to the academicians’ intentions to stay at their educational organizations and which finally leads to the development of their turnover intentions. 

The present study measured how cognitive distraction mediated the connection between despotic leadership and employee turnover intention. The outcomes of this study show that cognitive distraction positively mediated the connections between despotic leadership and employee turnover intention, thereby affirming hypothesis H5. This result corroborated the research, Naseer, et al. [104] despotic leadership in tandem with cognitive distraction exerts a positive effect on employee turnover intention. Similarly, Naseer, Raja, Syed, Donia and Darr [104] suggested exploring despotic leadership to offer a holistic understanding of the adverse outcomes of despotic leadership behavior. Accordingly, Syed, et al. [105] found that despotic leadership negatively affects employee performance and positively affects employee turnover intention in the service sector in Pakistan. Therefore, the present study result adds to the body of knowledge regarding the role cognitive distraction plays in the positive mediation of the relationship between despotic leadership and employee turnover intention in Chinese educational institutions. Thus, despotic leadership creates cognitive distraction in academicians’ work, which ultimately leads them to decide to leave their educational organizations. 

Finally, this study found that age and experience positively and significantly affect employee turnover intention under the influence of despotic leadership and a toxic workplace environment. Therefore, we affirmed hypotheses H6a and H6b. Our study supports previous research results that show a positive relation between both experience Mo, et al. [106] and age Bellotti, et al. [107] and the turnover intention of the individual in a toxic workplace environment. It may be because employees with more experience know their fields and may find job opportunities in other organizations and other benefits or retire instead of continuing to work in a toxic workplace environment with cognitive distraction under the influence of despotic leadership. 

### 7.2. Practical Implications

Employee retention is considered a critical problem in contemporary organizations. The primary contribution of the present study is an exploration of the predictors of employee turnover intention, which is an area of high theoretical and practical significance. Although researchers have explored employee turnover intention in different organizational sectors in different areas of the world, the present study explored the direct relationships between despotic leadership, toxic workplace environment, and cognitive distraction, and their influence on employee turnover intention in a more limited environment. In addition, the roles of toxic workplace environment and cognitive distraction as mediators was also studied. 

The key implications of this study address employee turnover intention in contemporary academic organizations and suggest that organizational leadership must develop self-assessment mechanisms to identify when, why, and how leaders turn despotic. Second, university leadership must monitor the workplace environment and workplace distraction to keep employee turnover intention low. Third, human resource departments must promote leadership development to reap the long-term benefits of organizational sustainability and growth. They must also develop a general understanding of their members to enable them to identify and report incidents of despotic leadership, workplace toxicity, and cognitive distraction. Doing so will benefit both individuals and institutions. In this regard, organizational policies must be updated to minimize or neutralize the direct and indirect antecedents of employee turnover intention and to address despotic leadership, workplace toxicity, and cognitive distractions. 

This study contributes to the literature by generalizing the relationship between despotic leadership and employee turnover intention. It also opens up future avenues for testing this model in other organizational and cultural settings. Finally, it highlights three critical factors in employee turnover intention: despotic leadership, toxic workplace environment, and cognitive distraction, as well as their underlying relationships. The findings of this study will be of high significance for practitioners, policy makers, and researchers interested in employee retention, especially in the university setting. Practitioners can improve academic institutional environments by understanding leadership roles. Educational policymakers may set up assessment criteria for discouraging the induction of academic leaders, such as rectors, deans, or heads of departments, with despotic leadership styles. This would result in educational organizations capable of retaining employees and would encourage a healthy work environment where cognitive distraction was minimized.

## 8. Conclusions

For the present study, a model was developed utilizing insights from organizational leadership theory and the previous literature. A survey of faculty members of Chinese educational institutions was conducted to test the model using PLS–SEM. The findings of the study bring important consequences for practitioners, academics, and researchers. They highlight the dire need for examining despotic leadership, toxic workplace environment, cognitive distraction, and employee turnover intention in Chinese higher education institutions. Mainly, despotic leadership and employee turnover intention were found to have a direct, significant, and positive effect on employee turnover intention. Toxic workplace environment and cognitive distraction also positively influenced that relationship. Toxic workplace environment and cognitive distraction indirectly and significantly increased employee turnover intention, thereby mediating the relationship between despotic leadership and employee turnover intention.

Several conclusions can be drawn from the outcomes of the present study. First, despotic leadership is an antecedent of employee turnover intention at higher education institutions in China. Second, a toxic workplace environment is also a predictor of employee turnover intention. Third, cognitive distraction also predicts employee turnover intention. Fourth, a toxic workplace environment positively mediates the relationship between despotic leadership and employee turnover intention. Fifth, cognitive distraction amplifies the effects of despotic leadership on employee turnover intention. Finally, the relationship between despotic leadership and employee turnover intention is prevalent in Chinese academic institutions, as reported in different organizational sectors in previous studies.

### Limitations and Future Research

The current study contains limitations that may affect interpretation of the findings and generalizations. First, it was conducted entirely in the Chinese academic context and based on the responses of personnel working in Chinese higher education institutions, a factor that limits the interpretation and applicability of its conclusions. Empirical evidence from other countries may validate the results of the present study. Furthermore, the data were collected from teachers from only four faculties (life sciences, social sciences, business sciences, and applied sciences). Employees in other faculties and non-teaching employees were not included in the sample. It is also a limitation of the study that semi-structured interviews were not conducted to further explore the depths of the phenomenon. A mixed methods approach may validate the results of the study. It was a cross-sectional study. A longitudinal research design would have documented both employee turnover intention and turnover behavior. Future research may incorporate the responses of such individuals for more interesting and useful findings. Finally, future research may investigate the relationship between despotic leadership and employee well-being, with both petty tyranny and cognitive distraction functioning as mediators. It would also be interesting to see the relationship between leadership, the work environment, and employee performance contribute to creating a guide to good practices in the field.

## Figures and Tables

**Table 1:** Participants profiles.

Characteristics	Category	Frequency (*f*)	Percentage (*%*)
Gender	Male	133	55.4
	Female	107	44.6
	Total	240	100.0
Age Group (in years)	Less than 30	72	30.0
	30–40	132	55.0
	41–50	24	10.0
	51–60 and above	12	5.0
	Total	240	100.0
Qualifications	Master’s/MS/MPhil	72	30.0
	PhD	84	35.0
	Postdoctoratal	48	20.0
	Others	36	15.0
	Total	240	100.0
Experience (in years)	Less than 10	96	40.0
	11–20	37	15.4
	21–30	96	40.0
	31 and above	11	4.6
	Total	240	100.0
Designation	Lecturer	131	54.6
	Senior Lecturer	48	20.0
	Associate Professor	49	20.4
	Professor	12	5.0
	Total	240	100.0
Employment Status	Full-Time Permanent	108	45.0
	Part-Time Permanent	60	25.0
	Contractual	36	15.0
	Others	36	15.0
	Total	240	100.0
Faculty	Life Sciences	47	19.6
	Social Sciences	65	27.1
	Business Sciences	79	32.9
	Natural Sciences	49	20.4
	Total	240	100.0

**Table 2:** Reliability and convergent validity.

Dimensions of Constructs	Factor Loading	Cronbach’s Alpha	rho_A	Composite Reliability	Average Variance Extracted (AVE)
Despotic Leadership (DL)		0.916	0.978	0.981	0.896
DL1	0.937				
DL2	0.974				
DL3	0.961				
DL4	0.966				
DL5	0.888				
DL6	0.950				
Toxic Workplace Environment (TWE)		0.917	0.936	0.934	0.672
TWE1	0.748				
TWE2	0.841				
TWE3	0.831				
TWE4	0.905				
TWE5	0.836				
TWE6	0.648				
TWE7	0.901				
Cognitive Distraction (CD)		0.896	0.911	0.92	0.659
CD1	0.821				
CD2	0.677				
CD3	0.806				
CD4	0.853				
CD5	0.819				
CD6	0.880				
Employee Turnover Intention (ETI)		0.868	0.873	0.902	0.606
ITI1	0.798				
ITI2	0.736				
ITI3	0.795				
ITI4	0.868				
ITI5	0.808				
ITI6	0.650				

**Table 3:** Discriminant validity (HTMT).

Constructs	CD	DL	ETI	TWE
Cognitive Distraction (CD)	0.812			
Despotic Leadership (DL)	0.701	0.846		
Employee Turnover Intention (ETI)	0.727	0.822	0.779	
Toxic Workplace Environment (TWE)	0.768	0.781	0.837	0.820

**Table 4:** Collinearity and model fit.

Dimensions	VIF-CD	VIF-ETI	Model Fit
Despotic Leadership	1.000	2.894	SRMR: 0.079
Toxic Workplace Environment (TWE)		3.567	NFI: 0.87
Cognitive Distraction (CD)		2.683	RMS_Theta: 0.121

**Table 5:** R-squared.

Constructs	R-Squared	R-Squared Adjusted
Toxic Workplace Environment	0.609	0.608
Cognitive Distraction	0.491	0.489
Employee Turnover Intention	0.828	0.824

**Table 6:** Descriptive analysis.

Constructs	N	Minimum	Maximum	Mean	Std. Deviation
Despotic Leadership	240	1.00	7.00	5.372	1.263
Toxic Workplace Environment	240	1.00	7.00	4.273	1.325
Cognitive Distraction	240	1.00	7.00	4.629	1.216
Employee Turnover Intention	240	1.00	7.00	5.472	1.336

**Table 7:** Direct relations.

Direct Relations	Coefficients	Mean	SD	T Statistics	*p* Values	Results
DL -> ETI	0.330	0.329	0.053	6.177	0.000	Accepted
TWE -> ETI	0.533	0.534	0.05	10.588	0.000	Accepted
CD -> ETI	0.122	0.122	0.04	3.024	0.003	Accepted
Age -> ETI	0.136	0.329	0.053	6.177	0.000	Accepted
Experience -> ETI	0.207	0.534	0.05	10.588	0.000	Accepted

Note: DL, despotic leadership; ETI, employee turnover intention; TWE, toxic workplace environment; CD, cognitive distraction.

**Table 8:** Indirect relations.

Direct Relations	Coefficients	Mean	SD	T Statistics	*p* Values	Results
DL -> TWE -> ETI	0.416	0.418	0.046	9.006	0.005	Accepted
DL -> CD -> ETI	0.086	0.086	0.028	3.034	0.019	Accepted

Note: DL, despotic leadership; ETI, employee turnover intention; TWE, toxic workplace environment; CD, cognitive distraction.

## Data Availability

The authors will provide data on reasonable request.
